# IL‐10‐secreting engineered macrophages alleviate DSS‐induced colitis with macrophage phenotypic modulation and gut microbiota remodeling in mice

**DOI:** 10.1002/btm2.70178

**Published:** 2026-10-22

**Authors:** Hanfei Liu, Junhua Liu, Shuhuang Peng, Jiwei Zhao, Fumeng Yang, Linwei Zhang, Shufang Wang, Luo Zhang, Gang Sun

**Affiliations:** ^1^ Chinese PLA Medical School Beijing China; ^2^ Department of Gastroenterology and Hepatology The First Medical Center of PLA General Hospital Beijing China; ^3^ Research Center of Bioengineering, The Medical Innovation Research Division of PLA General Hospital Beijing China; ^4^ The Medical School of Nankai University Tianjin China

**Keywords:** colonic macrophages, engineered macrophages, gut microbiota, IL‐10, ulcerative colitis

## Abstract

Ulcerative colitis (UC) is a chronic recurrent inflammatory bowel disease characterized by abdominal pain, hematochezia, and weight loss. Although remarkable progress has been made toward understanding the pathogenesis of UC, finding more effective treatments for this disease remains a challenge. Interleukin‐10 (IL‐10) possesses potent anti‐inflammatory properties, yet its therapeutic application in UC has failed clinically due to poor targeting and a short half‐life. In this study, we developed IL‐10‐secreting engineered macrophages (IL‐10M) based on RAW264.7 cells and evaluated their therapeutic effects in a dextran sulfate sodium‐induced colitis model. Intraperitoneal administration of IL‐10M alleviated body weight loss, disease activity, colon shortening, and histological injury. IL‐10M also reduced mucosal damage and improved tight junction‐associated epithelial barrier markers. Flow cytometry further detected engineered macrophage‐associated GFP signals in colonic tissues and revealed an early increase in CD206^+^ endogenous colonic macrophages. Microbiome profiling showed treatment‐associated compositional changes, including alterations in *Bacteroides acidifaciens* and *Mucispirillum schaedleri*, indicating microbiota remodeling after engineered macrophage treatment. Short‐term safety assessment did not reveal overt hematological abnormalities or major histological injury in liver and spleen. Overall, these findings suggest that IL‐10‐secreting engineered macrophages may represent a promising macrophage‐based strategy for mitigating intestinal inflammation in experimental colitis.

**Translational Impact Statement**

This study engineered macrophages to overexpress the anti‐inflammatory cytokine IL‐10 and evaluated their therapeutic potential in DSS‐induced colitis. IL‐10M alleviated disease activity, mucosal injury, and epithelial barrier disruption, with detectable IL‐10M‐associated GFP signals in the colonic macrophage compartment and accompanying changes in endogenous macrophage‐associated phenotypes and gut microbiota composition. These findings suggest that macrophage‐mediated IL‐10 delivery may provide a promising immunomodulatory strategy for experimental intestinal inflammation.

## 1 INTRODUCTION

Ulcerative colitis (UC) is a chronic recurrent inflammatory bowel disease (IBD) characterized by abdominal pain, hematochezia, and weight loss.^1^ In recent years, the incidence and prevalence of UC are increasing worldwide, imposing a huge burden on public health.^2^, ^3^ Although the precise etiology of UC has not been fully elucidated, it is widely believed to result from an uncontrolled immune response to various factors—including environmental, epithelial, microbial, and immune‐related triggers—in genetically susceptible individuals.^1^, ^2^, ^4^ The intestinal immune system maintains tolerance to dietary and gut microbiota antigens through sophisticated regulatory mechanisms, while simultaneously mounting appropriate immune responses against pathogens to preserve intestinal homeostasis.^5^ Cytokine‐mediated intercellular communication plays a fundamental role in this precise regulatory network. However, disruption of this balance—driven by elevated pro‐inflammatory cytokines (e.g., TNF‐*α*, IL‐6, IL‐1*β*, IL‐23) and impaired anti‐inflammatory mechanisms (functional regulatory T cells and associated anti‐inflammatory cytokines such as IL‐10 and TGF‐*β*)—can lead to chronic inflammatory disorders and tissue damage in the intestine.^6^ IL‐10, a multi‐source, pleiotropic cytokine with potent anti‐inflammatory properties, is crucial for intestinal homeostasis.^7^, ^8^ It functions as a master regulator of the intestinal anti‐inflammatory response by suppressing the production of pro‐inflammatory cytokines and chemokines, promoting the secretion of anti‐inflammatory mediators, and inhibiting the activation of naïve CD4^+^ T cells as well as the activation and proliferation of macrophages.^9^ The critical nature of IL‐10 signaling is underscored by the findings that mice harboring genes deficient in IL‐10 or IL‐10 receptor develop severe spontaneous colitis.^10^, ^11^ This conclusion was further validated in humans, as neonates with defective IL‐10 signaling pathway develop severe enterocolitis shortly after birth.^12^, ^13^, ^14^

Numerous preclinical studies support the potential of human IL‐10 (hIL‐10) in mitigating intestinal inflammation in IBD animal models. However, clinical trials of hIL‐10 for IBD have yielded disappointing outcomes.^15^, ^16^ Possible reasons include: the failure to achieve effective concentrations of hIL‐10 in intestinal tissues and the counteraction of its immunosuppressive properties by concurrent immunostimulatory effects, both of which are consequences of systemic administration. Therefore, developing therapies that enable targeted delivery of IL‐10 to inflamed intestinal tissue is a critical strategy.

Macrophages, as central components of the innate immune system, are pivotal guardians of intestinal homeostasis. However, in the disease context of IBD, specific macrophage populations can promote inflammatory responses and drive tissue damage.^17^, ^18^ This plasticity makes them compelling therapeutic targets in IBD. In recent years, engineered immune cell therapies have emerged as a transformative approach in precision medicine. Macrophages, with their innate capacity for inflammatory chemotaxis, represent ideal living vehicles for targeted delivery of therapeutic molecules.^19^, ^20^

Based on this rationale, we hypothesized that engineered macrophages secreting IL‐10 could serve as a targeted therapy to ameliorate colitis. In this study, we generated IL‐10‐secreting engineered macrophages (IL‐10M) and evaluated their therapeutic potential in a mouse model of dextran sulfate sodium (DSS)‐induced colitis. We further investigated their effects on endogenous colonic macrophage‐associated phenotypes and gut microbiota composition. Our results demonstrate that IL‐10M therapy significantly alleviates intestinal inflammation, supporting this cell‐based approach as a promising therapy for ulcerative colitis.

## 2 RESULTS

### 2.1 IL‐10M alleviates DSS‐induced colitis in mice

The IL‐10M cells used in this study were generated using the same lentiviral engineering strategy as previously described.^20^ Before animal administration, IL‐10 secretion was quantified in vitro to ensure functional activity (Figure S1). To evaluate the therapeutic effect of IL‐10M on UC, we established a DSS‐induced colitis mouse model and administered the corresponding treatment intraperitoneally at the onset of DSS exposure (Figure 1a). The model group (DSS + PBS group) exhibited marked weight loss, diarrhea, and hematochezia compared with controls, indicating successful induction of colitis. IL‐10M treatment effectively alleviated these symptoms, as reflected by stable body weight, more formed stool, and lower DAI scores (Figure 1b,d). IL‐10M treatment also effectively restored colon length compared with the model group (5.35 ± 0.32 cm), with no significant difference between the IL‐10M group (7.42 ± 0.47 cm) and the control group (7.67 ± 0.39 cm) (Figure 1c,e). ConM treatment partially ameliorated colon shortening (6.25 ± 0.47 cm), but failed to alleviate weight loss and rectal bleeding in DSS‐treated mice.

**FIGURE 1:**
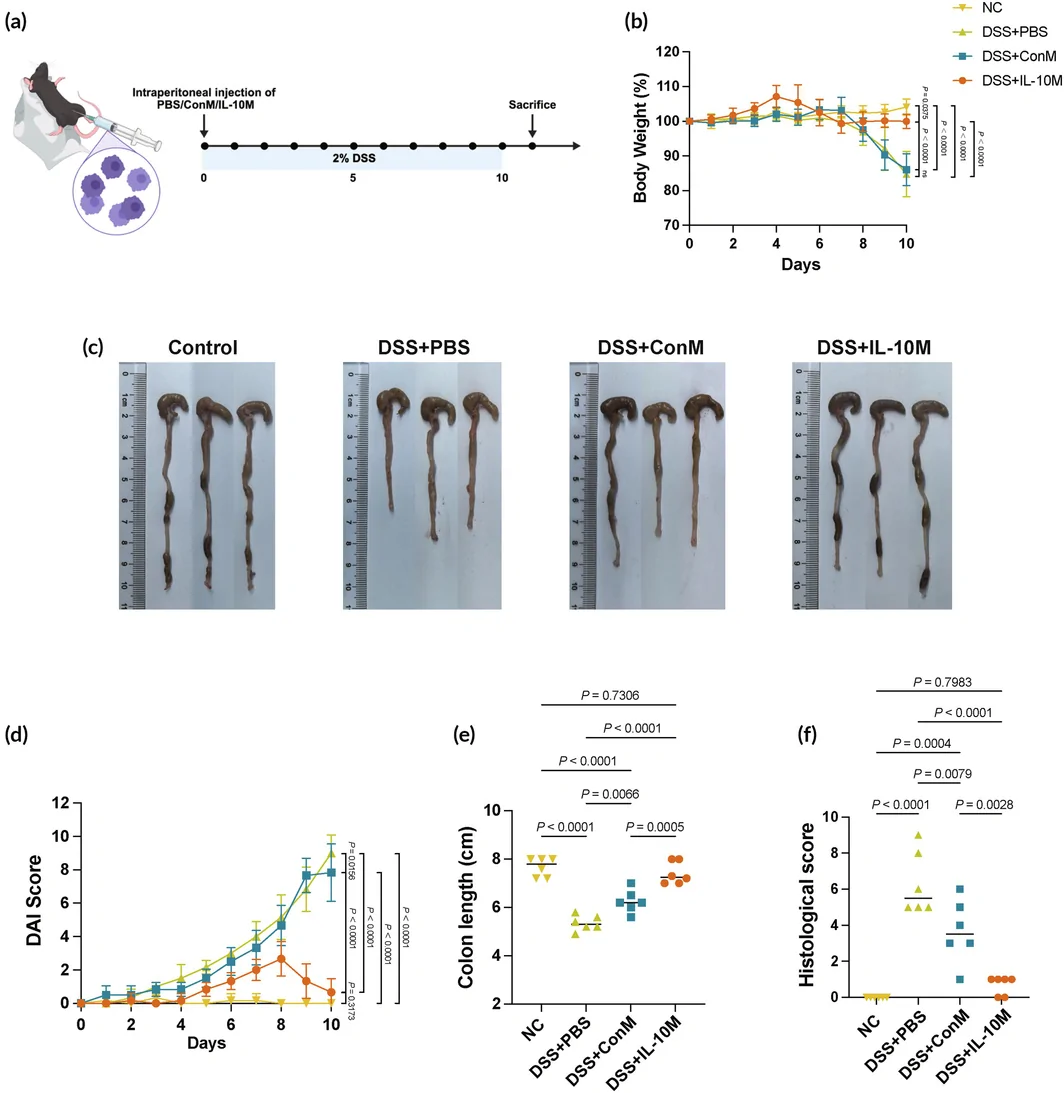
IL‐10M alleviates the symptoms of DSS‐induced colitis in mice. (a) Overview of experimental groups and timelines. (b) Body weight of mice during the treatment. (c) Photographs of the dissected cecum and colon tissues of mice in different groups. (d) Disease activity index (DAI) score. (e) Colon length of mice in different groups (*n* = 6). (f) Histological score of colon tissue section. Data are mean ± SD (*n* = 6).

Importantly, post hoc comparisons between ConM and IL‐10M were perfomed for all major efficacy endpoints. Compared with ConM, IL‐10M produced greater overall improvement, as reflected by better body weight recovery, reduced DAI scores, restored colon length, and lower histological scores. These findings indicate that sustained IL‐10 expression contributes substantially to the therapeutic efficacy of engineered macrophages in DSS‐induced colitis.

We performed Hematoxylin and Eosin (H&E) staining of colon tissue to evaluate the effect of IL‐10M treatment and quantified the severity of colitis through histological scores (on a scale of 0–11) based on the severity of inflammation, crypt damage, and ulceration.^21^ The pathological examination of colon tissues revealed that DSS administration resulted in infiltration of inflammatory cells into the mucosa and submucosa, entire crypt loss with multiple ulceration (Figure 2a). The IL‐10M intervention significantly alleviated inflammatory cell aggregation and maintained the integrity of colon tissue, with a significantly lower histological score (0.67 ± 0.52) compared to the model group (6.33 ± 1.75) (Figure 1f). Although the intervention of ConM partially preserved crypt structure, it failed to attenuate the infiltration of inflammatory cells. Quantification of colonic inflammatory cytokines by ELISA showed that IL‐10M administration resulted in a non‐significant elevation of IL‐10 level, while causing significant decreases in IL‐1*β* and IL‐6 (Figure 2b–d). TNF‐*α* expression exhibited a non‐significant downward trend (Figure 2e).

**FIGURE 2:**
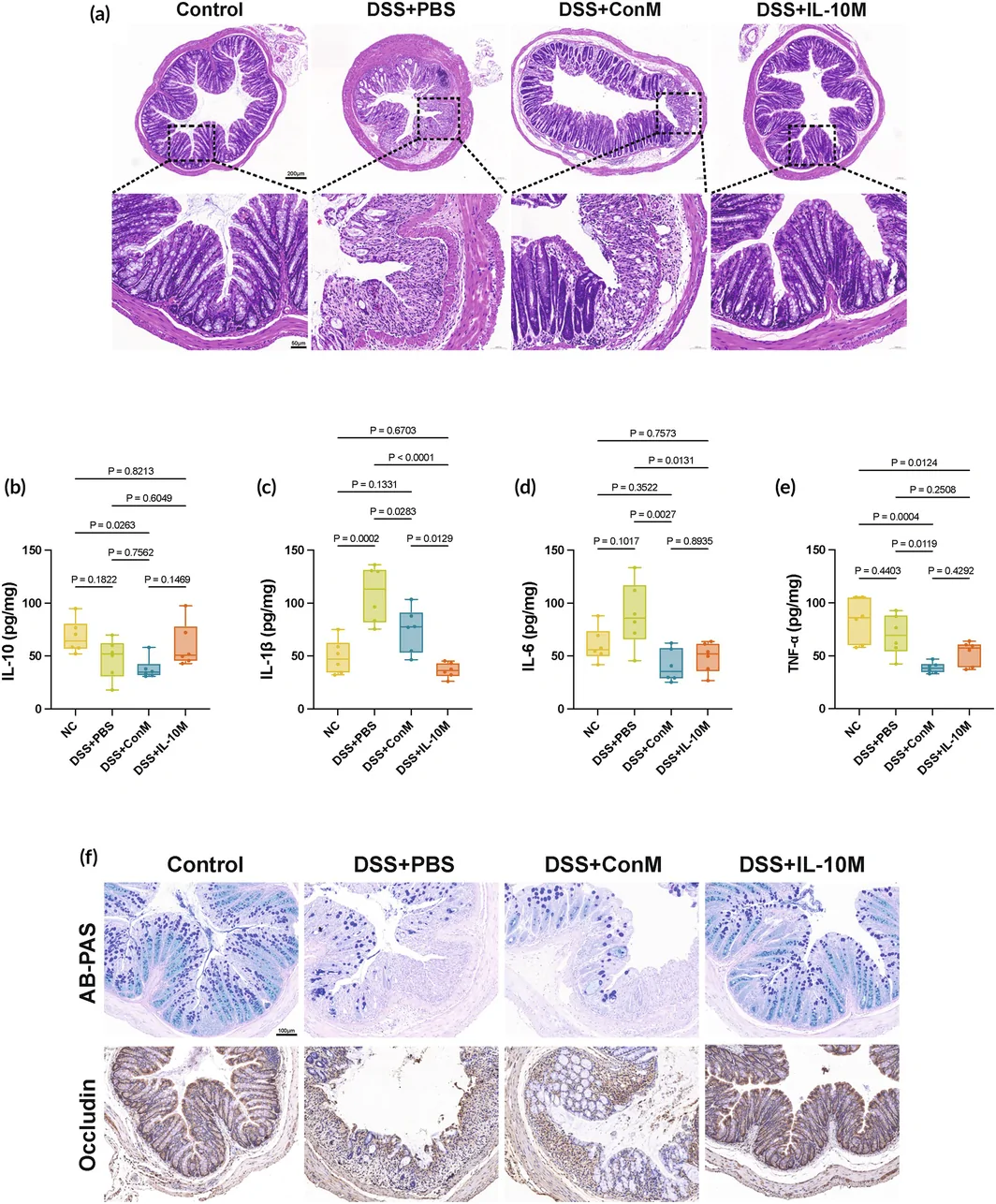
Ameliorative effect of IL‐10M on colonic inflammation and tissue damage. (a) Low‐magnification (top) and high‐magnification (bottom) H&E staining of colon tissues of mice in different groups. The scale bars are 200 and 50 μm for top and bottom, respectively. (b) IL‐10, (c) IL‐1*β*, (d) IL‐6, (e) TNF‐*α* concentrations in colonic tissue measured by ELISA. Data are mean ± SD (*n* = 6). (f) Representative pictures of immunohistochemical staining of Occludin and AB‐PAS staining of colon sections. The scale bars are 100 μm.

### 2.2 Protective effect of IL‐10M on epithelial barrier integrity

The intestinal mucosal barrier is an important defense system composed of mechanical, chemical, biological, and immune barriers, and it plays a crucial role in maintaining intestinal homeostasis and health. However, ulcerative colitis is characterized by dysfunction of the intestinal mucosal barrier.^22^ To assess the intestinal barrier function, we performed AB‐PAS staining on colon tissues to evaluate mucus production and immunohistochemistry to examine the expression of tight junction proteins (Occludin).^23^ DSS induction led to a significant reduction in goblet cells and impaired mucus production compared to the controls (Figure 2f). The expression of Occludin was also downregulated in the models. As expected, IL‐10M intervention restored the population of goblet cells (Figure 2f), mucus production, and the expression of intestinal tight junction proteins, exhibiting high efficiency in maintaining intestinal mucosa integrity. No significant effect of ConM intervention on intestinal mucosal integrity was observed.

### 2.3 IL‐10M dynamically modulates endogenous colonic macrophage‐associated phenotypes

The intestinal macrophage pool consists of multiple transcriptionally distinct subsets that undergo dynamic shifts during intestinal homeostasis, active inflammation, and inflammatory resolution.^17^, ^18^ To further clarify the dynamic retention of engineered macrophages and determine whether IL‐10M affected endogenous intestinal macrophages, we performed additional flow cytometry on Day 3, Day 6, and Day 9 after engineered macrophages administration (Figure 3a).

**FIGURE 3:**
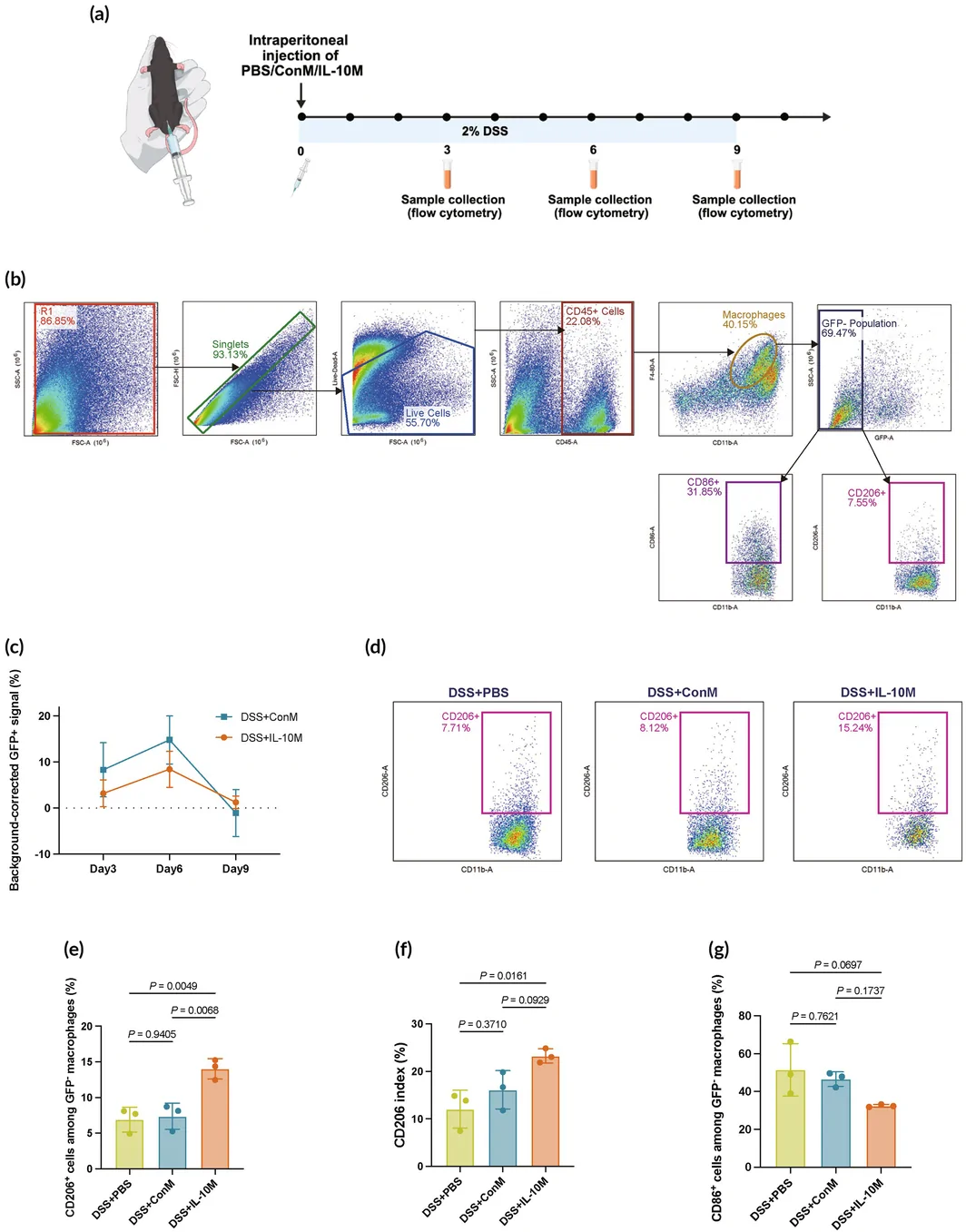
Detection of IL‐10M‐associated signals in colonic tissues and modulation of endogenous colonic macrophage‐associated phenotypes. (a) Schematic illustration of the experimental design for flow cytometry analysis. (b) Representative flow cytometry gating strategy. (c) Background‐corrected GFP^+^ macrophage‐associated signals in colonic tissues at different time points. Background correction was performed by subtracting the GFP^+^ signal detected in DSS + PBS mice at the corresponding time point. (d) Representative flow cytometry plots showing CD206 expression in GFP^−^ endogenous colonic macrophages on Day 3. (e) Quantification of CD206^+^ cells among GFP^−^ endogenous colonic macrophages on Day 3. (f) Quantification of the CD206 index [CD206%/(CD206% + CD86%)] in GFP^−^ endogenous colonic macrophages on Day 3. (g) Quantification of CD86^+^ cells among GFP^−^ endogenous colonic macrophages on Day 6. Data are presented as mean ± SD (*n* = 3).

Colonic lamina propria cells were first gated by FSC/SSC to exclude debris, followed by sequential selection of singlets, live cells, CD45^+^ leukocytes, and CD11b^+^F4/80^+^ macrophages. Because the administered engineered macrophages intrinsically expressed GFP, macrophages were further divided into GFP^+^ engineered macrophages and GFP^−^ endogenous macrophages (Figure 3b). CD86 and CD206 expression was then analyzed within the GFP^−^ macrophage population to evaluate phenotypic changes in endogenous colonic macrophages. After correction for background autofluorescence observed in DSS + PBS mice, GFP‐associated signals reached a relatively high level on Day 6 and then declined on Day 9 after ConM or IL‐10M administration (Figure 3c). These results suggest that engineered macrophage‐associated GFP signals were transiently detectable in colonic tissues during DSS‐induced colitis, but did not show sustained elevation.

Within the GFP^−^ endogenous macrophage population, we further analyzed the proportions of CD86^+^ and CD206^+^ cells. IL‐10M administration increased the proportion of CD206^+^ cells and the CD206 index at the early phase of DSS‐induced colitis (Day 3) (Figure 3d–f). GFP^−^CD86^+^ cells showed a decreasing trend after IL‐10M administration on Day 6, although the difference did not reach statistical significance (Figure 3g). These findings suggest that IL‐10M does not simply induce a sustained M2‐like shift, but dynamically modulates endogenous colonic macrophage‐associated phenotypes during different phases of DSS‐induced colitis. Immunofluorescence staining further showed a tendency toward reduced iNOS‐associated signals in colonic tissues after IL‐10M treatment (Figure S2A).

### 2.4 Safety assessment of engineered macrophage treatment

We collected mouse blood samples to investigate the potential impact of macrophage therapy on key hematological parameters (Figure 4a–h). The results demonstrated that IL‐10M therapy significantly ameliorated anemia in UC mice, as evidenced by increased red blood cell (RBC) counts and hemoglobin (Hb) levels. No significant differences in platelet counts were observed among the groups. Although the total white blood cell (WBC) counts in all experimental groups fell within the normal range, the model group exhibited a trend toward leukopenia. IL‐10M treatment increased the WBC count, albeit not significantly. Furthermore, the IL‐10M‐treated group showed an elevation in blood monocytes, which remained within the normal reference range. Neutrophil and lymphocyte counts were also within normal limits across all groups.

**FIGURE 4:**
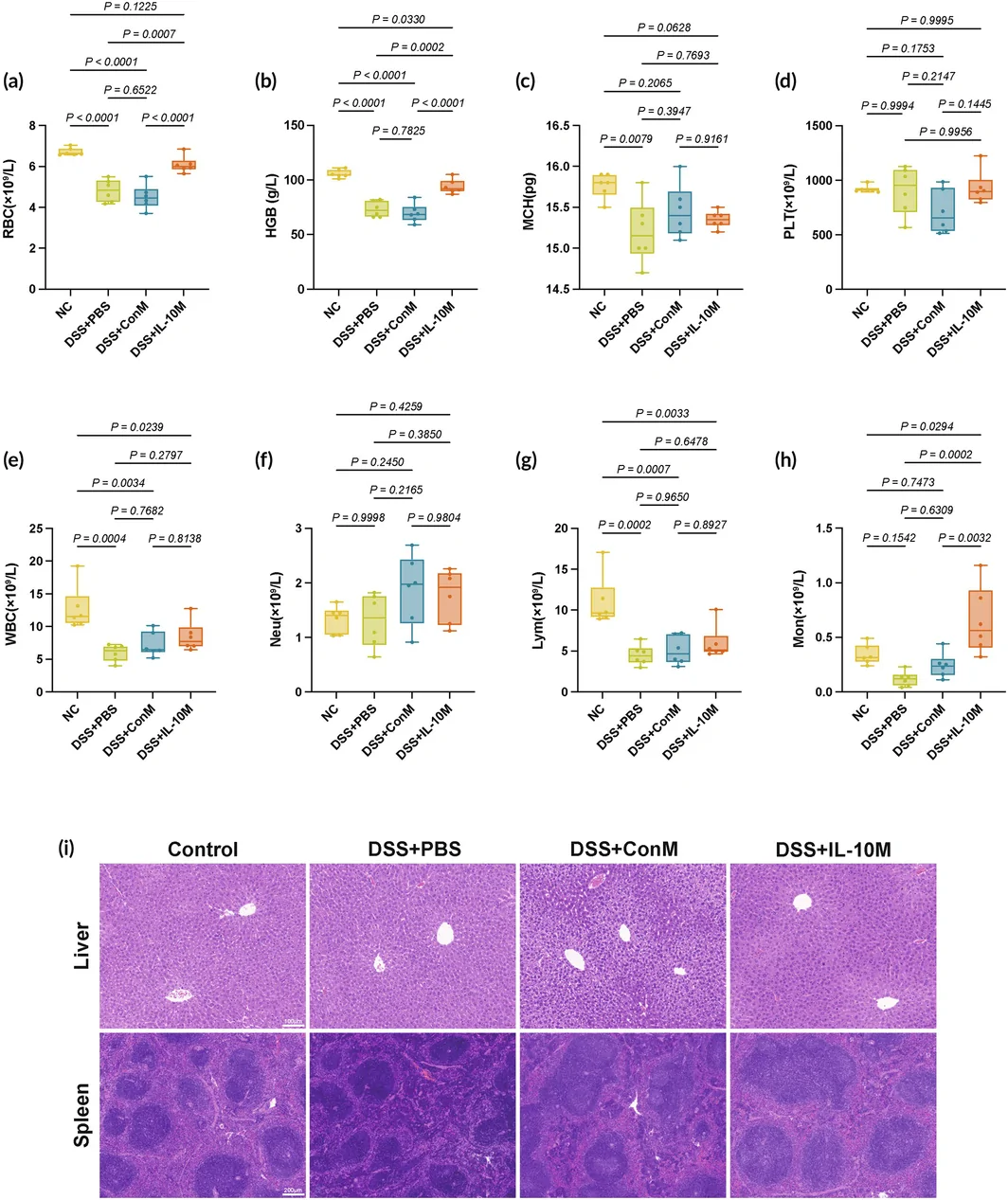
Safety assessment of engineered macrophage treatment. (a)–(h) Analysis of key hematological parameters of mice in each group. (i) H&E staining of liver and spleen tissues of mice in different groups. The scale bars are 100 and 200 μm, respectively. Data are mean ± SD (*n* = 6).

At necropsy, no gross abdominal masses, tumor‐like nodules, obvious organ enlargement, or macroscopic abnormalities were observed in IL‐10M‐treated mice during the short‐term observation period. We further conducted histological analysis of the main abdominal organs to evaluate the safety of engineered macrophage treatment. The histological analysis of liver and spleen in IL‐10M intervention group showed no significant differences compared to the controls (Figure 4i). In the ConM treatment group, mild hepatocyte edema was observed in some of the liver tissue samples, but no signs of hepatocyte necrosis, inflammatory cell infiltration, or other obvious pathological abnormalities were observed (Figure 4i).

### 2.5 Engineered macrophage treatment is associated with compositional changes in gut microbiota

To characterize whether IL‐10M treatment was accompanied by changes in gut microbiota composition, we performed metagenomic sequencing of colonic contents. As shown in Figure 5a–d, there was a significant reduction in the richness of gut microbiota in the model group compared to the control group. Although IL‐10M treatment exhibited an upward trend in gut microbiota richness compared to the model group, the differences had no statistical significance. Evenness (Pielou's evenness index) of the gut microbiota was significantly increased in the model group, while markedly decreased following IL‐10M administration. However, the Shannon and Simpson index, reflecting both richness and evenness of gut microbiota, showed no statistical significance among the groups. The *β*‐diversity of intestinal flora of UC mice was measured by principal component analysis (PCA analysis) and non‐metric multidimensional scaling analysis (NMDS). The results revealed clear separation among the microbial communities of the control and DSS groups, while both ConM and IL‐10M treatment showed partial shifts in microbial composition relative to the DSS group (Figure 5e,f).

**FIGURE 5:**
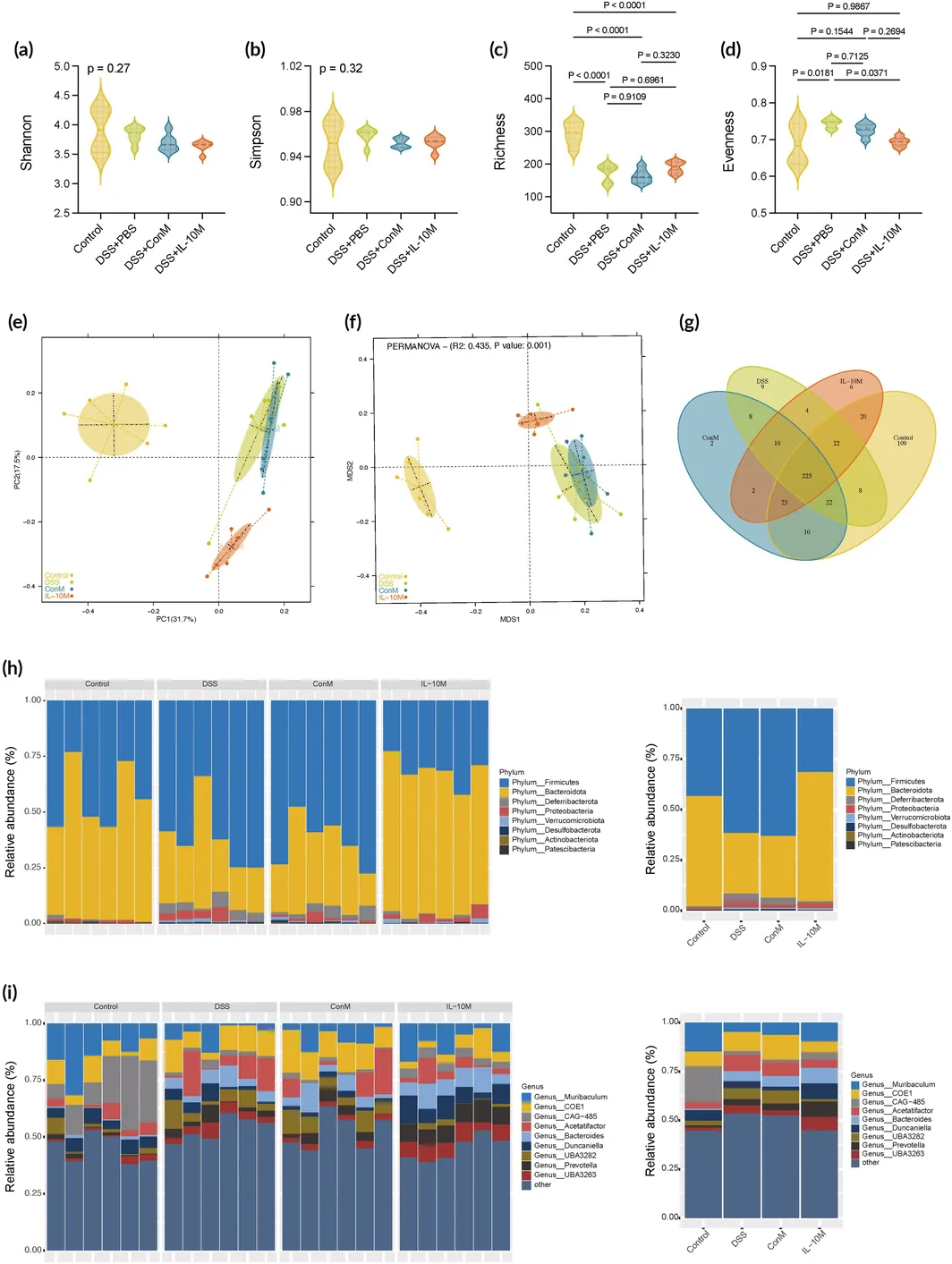
Engineered macrophage treatment is associated with changes in gut microbiota composition. (a)–(d) The *α*‐diversity index, including Richness (a), Evenness (b), Shannon index (c), and Simpson index (d) among different groups. (e) Principal component analysis (PCA). (f) Non‐metric multidimensional scaling (NMDS) analysis. (g) Venn diagram analysis of bacterial species of each group. (h) Relative abundance of gut microbiota structure at the phylum level. (i) Relative abundance of gut microbiota structure at the genus level. Data are mean ± SD (*n* = 6).

To compare the shared and unique features of gut microbiota composition among the four groups, a Venn diagram was constructed. The numbers of bacterial species in each group were as follows: Control group, 439; DSS group, 308; ConM group, 302; IL‐10M group, 312. The numbers of shared species between groups were: 277 between Control and DSS groups; 280 between Control and ConM groups; and 290 between Control and IL‐10M groups (Figure 5g). Gut microbiota composition was further compared at the phylum and genus levels. At the phylum level, the model and ConM groups showed an increase in the relative abundance of *Firmicutes* and a decrease in that of *Bacteroidota*, whereas the IL‐10M treatment group exhibited the opposite trend (Figure 5h). At the genus level, the model group displayed a decrease in the relative abundance of *Muribaculum* and *CAG‐485*, and an increase in that of *Acetatifactor* and *UBA3282*. In contrast, the IL‐10M group showed an increase in the relative abundance of *UBA3263*, *Prevotella*, and *Duncaniella*, and a decrease in that of *COE1*, *UBA3282*, and *Acetatifactor* (Figure 5i).

To identify bacterial taxa that differed among groups, we performed linear discriminant analysis effect size (LEfSe) analysis. As shown in Figure 6a, the top 10 species with differential abundance in each group were identified, including several taxa closely related to intestinal health and inflammation, such as *Muribaculum faecis*, *Paramuribaculum intestinale*, and *Muribaculum intestinale* in the control group, *Mucispirillum schaedleri* in the model group, and *Bacteroides acidifaciens* and *Duncaniella freteri* in the IL‐10M group. Notably, *B. acidifaciens*, a commensal bacterium reported to be involved in metabolic regulation, liver injury, and intestinal health,^24^, ^25^ was increased in the IL‐10M group compared with DSS + PBS mice, although the difference between ConM and IL‐10M did not reach statistical significance (Figure 6b). *M. schaedleri*, a mucus‐dwelling pathobiont,^26^, ^27^ was enriched in DSS‐treated mice but was significantly reduced after IL‐10M treatment compared with ConM (Figure 6c). Moreover, *M. intestinale*, a potential indicator of intestinal homeostasis,^28^ was depleted in DSS‐treated mice and showed partial recovery after engineered macrophage treatment, with no significant difference between ConM and IL‐10M (Figure 6d). IL‐10M treatment also increased the relative abundance of *D. freteri*, although its functional role remains poorly understood and the ConM–IL‐10M difference did not reach statistical significance (Figure 6e). Collectively, these results indicate that IL‐10M intervention was accompanied by compositional remodeling of the gut microbiota in DSS‐induced colitis. However, because several representative taxa did not differ significantly between ConM and IL‐10M, part of the microbiota remodeling may be attributable to the macrophage vehicle itself, with sustained IL‐10 expression providing additional effects in selected taxa.

**FIGURE 6:**
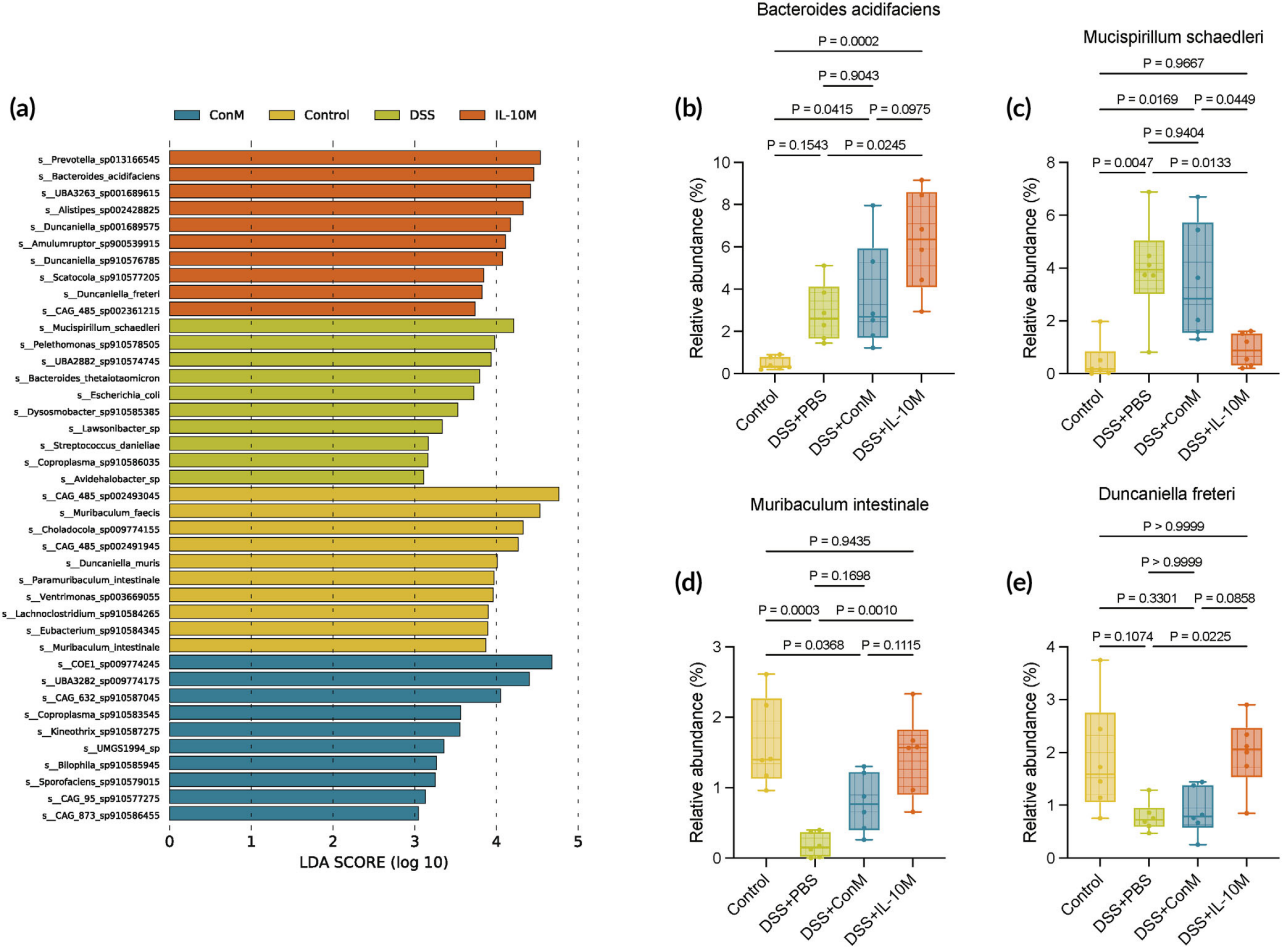
Identification and validation of representative bacterial species associated with engineered macrophage treatment. (a) LDA analysis comparing intestinal microbiota between groups at the species level. (b)–(e) Relative abundance of *Bacteroides acidifaciens* (b), *Mucispirillum schaedleri* (c), *Muribaculum intestinale* (d), and *Duncaniella freteri* (e) among different groups. Data are mean ± SD (*n* = 6).

## 3 DISCUSSION

In this study, we investigated the therapeutic potential of IL‐10‐secreting engineered macrophages in DSS‐induced colitis. Our results demonstrate that IL‐10M administration significantly alleviated weight loss, disease activity, colon shortening, histological injury, and epithelial barrier disruption. Although ConM intervention did not significantly improve weight loss, it showed partial effects on disease activity and colon damage. Importantly, compared with ConM, IL‐10M produced significantly greater improvement across the analyzed efficacy endpoints, supporting an important contribution of sustained IL‐10 secretion to the therapeutic activity of engineered macrophages.

Previous studies have explored strategies for the targeted intestinal delivery of IL‐10 to treat UC. For example, rectal administration of gelatin microspheres containing IL‐10^29^ and oral delivery of engineered probiotics^30^, ^31^ secreting IL‐10 have demonstrated efficacy in alleviating intestinal inflammation in animal models. Fay et al.^32^ developed a strategy using a fragment of non‐toxigenic *Vibrio cholerae* exotoxin as a carrier to deliver IL‐10 (termed AMT‐101) to the intestinal lamina propria. However, AMT‐101 lacks active targeting to inflammatory sites, affecting the entire intestinal tract instead. Liu et al.^33^ reported the use of galactose‐modified, chitosan/alginate hydrogel‐coated extracellular vesicles (termed Gal‐IL10‐EVs) for IL‐10 delivery. While Gal‐IL10‐EVs have the ability of macrophage targeting, they are substantially excreted after 48 h of oral administration, resulting in a limited duration of IL‐10 action.

The engineered macrophage (IL‐10M) strategy employed in this study offers several distinct advantages. First, macrophages possess intrinsic inflammatory chemotaxis, enabling their active recruitment to inflamed sites. Second, as potent antigen‐presenting and immunomodulatory cells, macrophages are not merely passive carriers; they actively phagocytose senescent cells, cellular debris, and pathogens, and are also a major physiological source of IL‐10 and other immunoregulatory cytokines.^34^ These inherent properties may integrate inflammatory tropism, local cytokine delivery, and intrinsic immunomodulatory capacity into a single cellular platform, making IL‐10M a promising macrophage‐based therapeutic approach.

The flow cytometry data further provided dynamic evidence for the interaction between administered engineered macrophages and host colonic macrophages. After background correction, GFP‐associated signals in the colonic macrophage compartment increased after cell administration, peaked around Day 6, and declined toward baseline by Day 9. This dynamic pattern suggests transient detectability rather than persistent retention of administered engineered macrophages in colonic tissues. The late decline may partly reflect immune clearance of RAW264.7‐derived cells in the allogeneic C57BL/6 recipient setting. In parallel, IL‐10M increased GFP^−^CD206^+^ endogenous macrophages and the CD206 index at Day 3, whereas GFP^−^CD86^+^ endogenous macrophages showed a decreasing trend at Day 6. These findings suggest a time‐dependent modulation of host macrophage‐associated phenotypes rather than a simple, fixed M1‐to‐M2 conversion.

Microbiome profiling showed that IL‐10M treatment was accompanied by changes in several bacterial taxa, including increased *B. acidifaciens*, *M. intestinale*, and *D. freteri*, as well as reduced *M. schaedleri*, compared with DSS + PBS mice. These changes are biologically plausible because intestinal inflammation, barrier disruption, and microbial ecology are closely interconnected. However, direct comparisons between ConM and IL‐10M showed that only *M. schaedleri* differed significantly, whereas differences in *B. acidifaciens*, *M. intestinale*, and *D. freteri* did not reach statistical significance. This suggests that the macrophage vehicle itself may contribute substantially to part of the observed microbiota remodeling, while sustained IL‐10 expression may provide modest and/or additive effects rather than acting as the sole determinant of microbial changes. Nevertheless, the present study cannot determine whether microbiota changes directly contributed to disease improvement or occurred secondarily as a consequence of reduced inflammation and improved barrier integrity. Future fecal microbiota transplantation, antibiotic depletion and recolonization, or germ‐free mouse experiments will be required to test causality.

Notably, colonic IL‐10 levels measured by ELISA at the terminal time point showed only a non‐significant increase after IL‐10M treatment, despite significant suppression of IL‐1*β* and IL‐6. This apparent discrepancy may be partly related to the sampling time point. Cytokines were measured in whole‐colon homogenates on day 10, whereas flow cytometry showed that GFP‐associated signals in the colon had markedly declined by the late phase of observation. Therefore, locally secreted IL‐10 may have acted earlier or in a cell‐proximal manner that was not fully captured by bulk tissue ELISA at the terminal time point. In addition, RAW264.7 cells are derived from BALB/c mice, whereas the recipient mice in this study were C57BL/6 mice. The allogeneic setting may have facilitated immune clearance of administered RAW264.7‐derived macrophages, which could limit long‐term persistence and uncontrolled expansion. This feature may theoretically limit sustained persistence, although definitive evaluation of biodistribution, persistence, and tumorigenicity requires dedicated long‐term studies.

Despite the promising results, several limitations of this study should be acknowledged. First, while the DSS‐induced acute colitis model is widely used, it does not fully simulate the chronic and relapsing characteristics of human UC. Future validation of the long‐term efficacy and safety of IL‐10M in chronic colitis animal models is necessary. Second, macrophage phenotype was assessed using flow cytometry with CD86 and CD206, which does not define a complete macrophage polarization program. Future studies using broader marker panels, single‐cell transcriptomics, or sorted‐cell RNA‐seq would help resolve macrophage subset dynamics more accurately. Third, IL‐10M was generated from RAW264.7 cells, an immortalized murine macrophage‐like cell line. This cell system offers practical advantages for proof‐of‐concept development, including stable expansion, controllable engineering, and reproducible IL‐10 expression. However, future studies using primary bone marrow‐derived macrophages, induced pluripotent stem cell‐derived macrophages, or other clinically relevant macrophage sources, together with long‐term safety monitoring, will be necessary to advance this platform toward translational application. Finally, the present IL‐10M system constitutively secretes IL‐10. The timing and magnitude of IL‐10 secretion cannot be dynamically adjusted according to the inflammatory microenvironment.^35^ To address this issue, inflammation‐responsive macrophages capable of demand‐adapted IL‐10 secretion may represent an important direction for future optimization.

## 4 CONCLUSION

In conclusion, IL‐10‐secreting engineered macrophages effectively alleviated DSS‐induced colitis in mice and were associated with improved epithelial barrier‐related markers, modulation of endogenous colonic macrophage‐associated inflammatory phenotypes, and changes in gut microbiota composition. This approach showed a favorable short‐term safety profile and may represent a promising macrophage‐based immunomodulatory strategy for the intervention of intestinal inflammation.

## 5 MATERIALS AND METHODS

### 5.1 Cell preparation

IL‐10M and ConM cells were generated using previously established lentiviral expression vectors in our laboratory, following the strategy described in our previous work.^20^ Briefly, the murine IL‐10 coding sequence was cloned into the pCDH‐MSCV‐MCS‐EF1*α*‐GFP‐T2A‐Puro vector, in which GFP served as a reporter for cell tracking and puromycin resistance was used for stable cell selection. An empty‐vector construct expressing GFP alone was used to generate ConM. Lentiviral particles were produced in 293FT cells using a standard third‐generation packaging system and subsequently used to transduce RAW264.7 macrophages. After puromycin selection, stable cell populations were established and expanded. Both IL‐10M and ConM constitutively expressed GFP.

RAW264.7 cell line was originally obtained from ATCC (American Type Culture Collection, TIB‐71™, RRID: CVCL_0493). All cells were cultured in RPMI 1640 medium (Gibco, C11875500BT) supplemented with 10% fetal bovine serum (ExCell, FND500) and 1% penicillin/streptomycin (Gibco, 15140‐122) and maintained at 37°C in a humidified 5% CO_2_ incubator.

For animal experiments, cells were expanded in 10 cm culture dishes. Prior to harvesting, 1 mL of culture supernatant was collected into a 1.5 mL microcentrifuge tube for IL‐10 quantification using a commercial Mouse IL‐10 ELISA kit (Boster, EK0417) according to the manufacturer's protocol. Subsequently, cells were washed once with PBS solution (Gibco, C10010500BT), detached, and centrifuged at 500 × *g* for 5 min. The cell pellet was resuspended in an appropriate volume of PBS and counted prior to in vivo administration. All cell lines above were grown under sterile conditions and periodically tested using a mycoplasma detection kit (Vazyme, D201) without contamination.

### 5.2 Mouse model and treatment protocol

All animal experiments were approved by the Experimental Animal Ethics Committee at the Chinese PLA General Hospital (approval number: SQ2024852). Eight‐week‐old male C57BL/6 mice were housed under specific pathogen‐free conditions. Prior to any experimental intervention, all mice underwent a minimum two‐week acclimatization period within the facility. Acute colitis was induced by administering 2% (w/v) DSS (molecular weight 36–50 kDa, MeilunBio) in drinking water ad libitum for 10 days. Age‐matched control mice received normal drinking water throughout the experiment.

Mice were randomly assigned to four groups (*n* = 6 per group): (1) Control group: normal water; (2) Model group: 2% DSS water + intraperitoneal (i.p.) injection of 200 μL sterile PBS; (3) ConM group: 2% DSS water + i.p. injection of 1 × 10^6^ ConM macrophages in 200 μL PBS; (4) IL‐10M group: 2% DSS water + i.p. injection of 1 × 10^6^ IL‐10M macrophages in 200 μL PBS. All cell or PBS treatments were administered on the first day of DSS exposure.

Body weight, stool consistency, and the presence of gross blood in feces or at the anus were monitored daily. The disease activity index (DAI) was calculated combining scores for weight loss (0–4), stool consistency (0–4), and bleeding (0–4).

Mice were fasted overnight on day 10. On the following day, blood samples were collected from the retro‐orbital plexus, after which animals were euthanized. The entire colon was excised from the cecum to the anus, and its length was measured. For histology, segments of the colon, spleen, and liver were fixed in 4% paraformaldehyde. A separate segment of colon was snap‐frozen in liquid nitrogen and stored at −80°C for inflammatory factor levels (TNF‐*α*, IL‐1*β*, IL‐6, and IL‐10) detection using ELISA kits. Colonic luminal contents were collected in DNA preserving fluid for microbial analysis.

### 5.3 Histological, immunofluorescence, and safety assessments

Paraffin‐embedded colon, liver, and spleen sections (5 μm) were stained with H&E. Histological scoring (0–11) of colon tissue^21^ was performed as previously described in a blinded manner based on the severity of inflammatory cell infiltration (0–3), crypt damage (0–5), and ulceration (0–3). Paraffin‐embedded colon sections were stained with Alcian Blue/Periodic Acid‐Schiff (AB‐PAS) to visualize goblet cells and mucin. The expression of tight junction protein Occludin in colon tissue was determined by immunohistochemistry. The expression of NOS2 and CD206 in colon tissue was evaluated by immunofluorescence staining. Complete blood count analysis was performed using a Mindray BC‐5000 Vet automated hematology analyzer (Mindray).

### 5.4 Flow cytometric analysis of colonic macrophages

To evaluate the distribution of administered engineered macrophages and their effects on endogenous colonic macrophage phenotypes, flow cytometric analysis was performed on colonic lamina propria cells collected on Days 3, 6, and 9 after intervention. Mice were randomly assigned to four groups: Control, DSS + PBS, DSS + ConM, and DSS + IL‐10M. The Control group was analyzed on Day 9 only, whereas DSS‐treated groups were analyzed at all three time points.

Briefly, freshly isolated colonic tissues were washed with sterile PBS, minced into small fragments, and digested in DMEM containing collagenase I (1 mg/mL), collagenase II (1 mg/mL), collagenase IV (1 mg/mL), neutral protease (0.5 mg/mL), and DNase I (200 μg/mL) at 37°C for 1 h with gentle agitation. The digestion was terminated with RPMI‐1640 medium supplemented with 10% fetal bovine serum. Cell suspensions were filtered through a 70‐μm cell strainer, centrifuged, and resuspended in PBS for counting.

For flow cytometric staining, cells were first incubated with Zombie R685 viability dye (BioLegend) to exclude dead cells, followed by Fc receptor blocking using anti‐mouse CD16/32 antibody (BioLegend). Surface staining was performed using antibodies against CD45, CD11b, F4/80, and CD86 (BioLegend). After fixation and permeabilization, intracellular staining was performed using anti‐CD206 antibody (BD). Samples were acquired on a Cytek NL‐CLC3000 spectral flow cytometer (Cytek Biosciences), and data were analyzed using FlowJo software (version 10.8). Antibodies and reagents used for flow cytometry are listed in Supplementary Table S1.

The gating strategy was as follows: debris was excluded based on FSC/SSC characteristics, followed by singlet selection, live‐cell gating, identification of CD45^+^ leukocytes, and subsequent gating of CD11b^+^F4/80^+^ macrophages. Because both ConM and IL‐10M constitutively expressed GFP, macrophages were further divided into GFP^+^ engineered macrophage‐associated cells and GFP^−^ endogenous macrophages. CD86 and CD206 expression was analyzed within the GFP^−^ macrophage population to evaluate phenotypic changes in endogenous macrophages.

The proportions of GFP^+^ macrophage‐associated cells, GFP^−^CD86^+^ macrophages, and GFP^−^CD206^+^ macrophages were quantified. In addition, a CD206 index was calculated as CD206%/(CD206% + CD86%) to reflect the relative predominance of CD206^+^ over CD86^+^ endogenous macrophages.

### 5.5 Gut microbiota metagenomic sequencing and bioinformatic analysis

Fecal sample preprocessing, metagenomic libraries construction, library quality control, and PE150 sequencing were all completed in Beijing QuantiHealth Technology Co., Ltd. (Beijing, China).

Clean Reads obtained after quality control were used as input data, and MetaPhlAn 4 was employed for taxonomic classification, thereby obtaining the relative abundance of the gut microbiota at various levels from phylum to species. Bioinformatic analysis of the metagenomic data was performed as follows. *α*‐diversity of the gut microbiota within each sample was assessed by calculating the Shannon, Simpson, species richness and Pielou's evenness indices using the diversity function in the R vegan package. PCA was conducted using the rda function in the R package. NMDS based on Bray–Curtis dissimilarity was performed using the metaMDS function. Permutational multivariate analysis of variance was employed to test for statistically significant differences in microbial species among the experimental groups.

A Venn diagram was constructed to visualize the shared and unique species across groups. Linear discriminant analysis (LDA) effect size (LEfSe) was applied to identify differences in the relative abundances of gut microbiota at the species level. LDA score >2.0 with a *p*‐value <0.05 was considered significantly enriched.

### 5.6 Statistical analysis

All analyses were performed using GraphPad Prism software (version 10.0). Data are presented as mean ± standard deviation (SD). Statistical significance among multiple groups was determined by one‐way ANOVA followed by Tukey's post hoc test, or by two‐way ANOVA for data with time factors. *p*< 0.05 was considered statistically significant.

## AUTHOR CONTRIBUTIONS

**Hanfei Liu:** Investigation; methodology; writing – original draft; writing – review and editing. **Junhua Liu:** Investigation; methodology; writing – original draft; writing – review and editing. **Shuhuang Peng:** Data curation; formal analysis. **Jiwei Zhao:** Formal analysis; data curation. **Fumeng Yang:** Investigation; data curation. **Linwei Zhang:** Investigation; data curation. **Shufang Wang:** Investigation; data curation. **Luo Zhang:** Project administration; supervision; writing – review and editing; funding acquisition. **Gang Sun:** Project administration; writing – review and editing; supervision; funding acquisition.

## CONFLICT OF INTEREST STATEMENT

The authors declare that they have no conflict of interest.

## Supporting information


Supplementary Figure S1.

Supplementary Figure S2.

**Supplementary Table S1.** Antibodies and reagents used for flow cytometry.

## Data Availability

The data that support the findings of this study are available from the corresponding author upon reasonable request.
