# Supplementary material for: IL‐10‐secreting engineered macrophages alleviate DSS‐induced colitis with macrophage phenotypic modulation and gut microbiota remodeling in mice
Source: Bioeng Transl Med. 2026 Oct 22:e70178. Online ahead of print. doi: 10.1002/btm2.70178 (PMC13687998; doi:10.1002/btm2.70178)
Supplement: Supplementary file 1 [file BTM2-9999-e70178-s001.pdf]

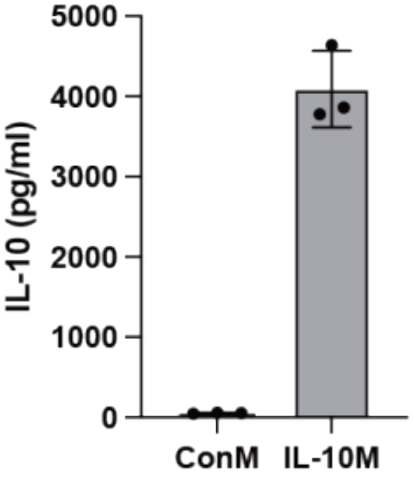

5000
4000
3000
2000
1000
0
IL-10 (pg/ml)
ConM
IL-10M
