# Supplementary material for: IL‐10‐secreting engineered macrophages alleviate DSS‐induced colitis with macrophage phenotypic modulation and gut microbiota remodeling in mice
Source: Bioeng Transl Med. 2026 Oct 22:e70178. Online ahead of print. doi: 10.1002/btm2.70178 (PMC13687998; doi:10.1002/btm2.70178)
Supplement: Supplementary file 2 [file BTM2-9999-e70178-s003.pdf]

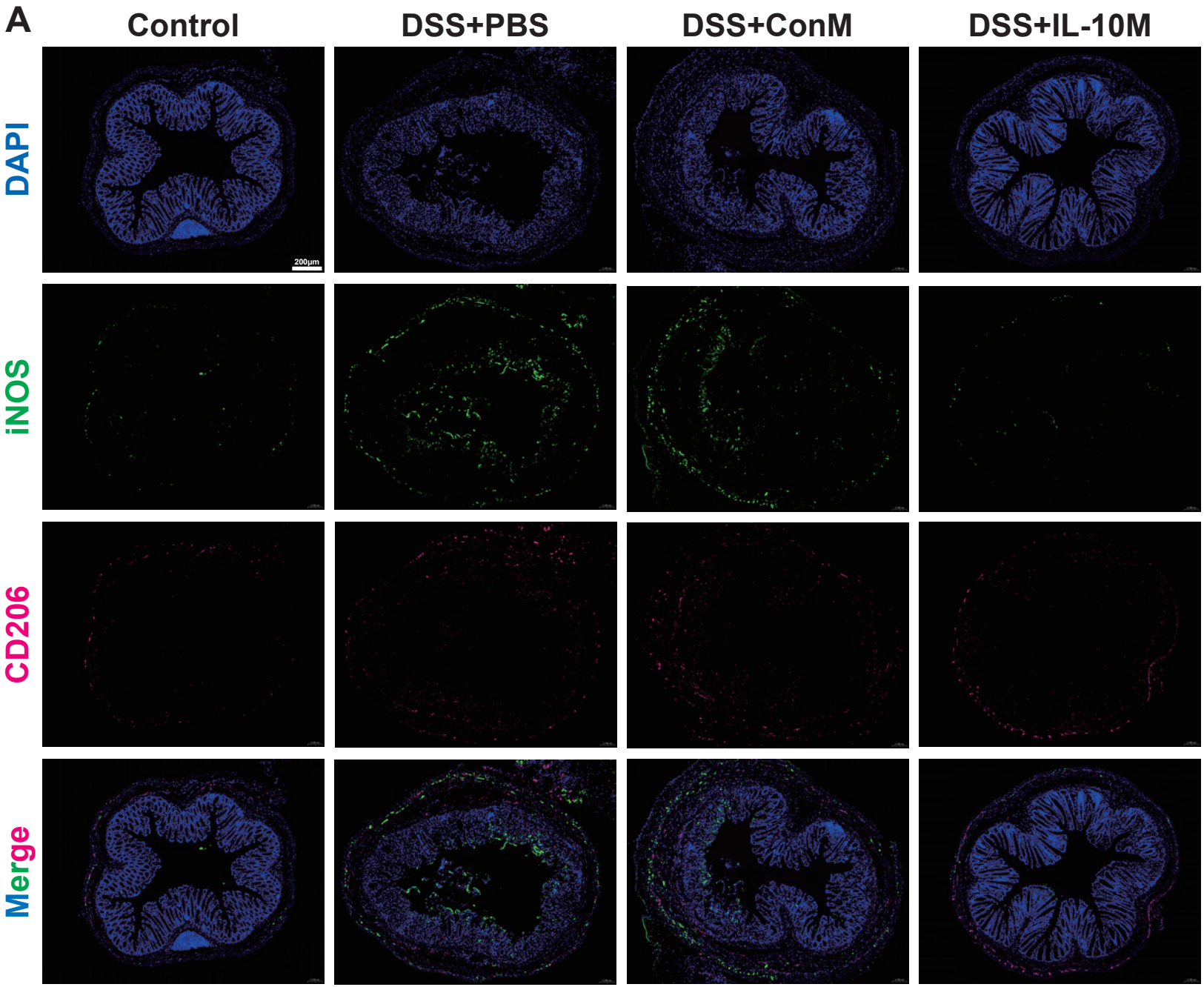
A
Control
DSS+PBS
DSS+ConM
DSS+IL-10M
DAPI
iNOS
CD206
Merge
200μm
