# Supplementary material for: IL‐10‐secreting engineered macrophages alleviate DSS‐induced colitis with macrophage phenotypic modulation and gut microbiota remodeling in mice
Source: Bioeng Transl Med. 2026 Oct 22:e70178. Online ahead of print. doi: 10.1002/btm2.70178 (PMC13687998; doi:10.1002/btm2.70178)
Supplement: Supplementary file 3 [file BTM2-9999-e70178-s002.docx]

Supplementary Table S1. Antibodies and reagents used for flow cytometry

| Marker | Fluorochrome | Catalog No. | Supplier |
| --- | --- | --- | --- |
| CD45 | PE/Dazzle 594 | 103146 | BioLegend |
| CD11b | APC/Cy7 | 101226 | BioLegend |
| F4/80 | PE/Cy7 | 123114 | BioLegend |
| CD86 | BV421 | 105032 | BioLegend |
| CD206 | PE | 141706 | BD |
| Zombie R685 | - | 423119 | BioLegend |
| anti-CD16/32 | - | 101320 | BioLegend |

Corresponding isotype controls (BioLegend 400549 and BD 400507) were used during antibody optimization and gating.
